# Changing definitions of disease: Transformations in the diagnostic criteria for Alzheimer's disease

**DOI:** 10.1002/alz.70133

**Published:** 2025-04-12

**Authors:** Lennart H. van der Molen, Marianne Boenink, Harro van Lente, Edo Richard

**Affiliations:** ^1^ IQ Health science department Radboudumc Nijmegen The Netherlands; ^2^ Society Studies department Maastricht University Maastricht The Netherlands; ^3^ Dept of Neurology, Donders Institute for Brain, Cognition and Behaviour Radboud University Medical Centre Nijmegen The Netherlands; ^4^ Dept of Public & Occupational Health Amsterdam University Medical Centre, University of Amsterdam Amsterdam The Netherlands

**Keywords:** Alzheimer's disease, Alzheimer's disease diagnosis, Alzheimer's disease diagnostic criteria, biomarkerization, biomarkers Alzheimer's disease, preclinical Alzheimer's disease, prodromal Alzheimer's disease, research in context

## Abstract

**INTRODUCTION:**

Whether Alzheimer's disease (AD) should be defined by symptoms, biological processes, or both, is a matter of debate. We aim to reconstruct the motivations, aims, and content of consecutive versions of AD diagnostic criteria.

**METHODS:**

We systematically analyzed publications on AD diagnostic criteria between 1984 and 2024.

**RESULTS:**

Early diagnosis and incorporating recent scientific findings are recurring aims for criteria revisions but aims and motivations for revising are often unclear or ambiguous and reflection on previous criteria is lacking. The subsequent criteria, except International Working Group (IWG) 2021/2024, consistently lower the threshold for diagnosing AD and increasingly focus on amyloid β and tau biomarkers.

**DISCUSSION:**

Subsequent AD criteria show an increasing “biomarkerization,” but it is often unclear what problems revised criteria should solve and how effective they are. To overcome these limitations, future revisions should evaluate the effectiveness and impacts of previous criteria, and define clear problems and aims.

**Highlights:**

- Early diagnosis and incorporating scientific insights are recurring aims.
- The aims of new criteria are often not clearly articulated or ambiguous.
- The number of requirements for an AD diagnosis decreases over time.
- Consecutive criteria for research and clinical use did not result in clear terminology.
- The AD definition is increasingly narrowed to amyloid β and tau.

## 1 BACKGROUND

The Alzheimer's Association (AA) recently published revised diagnostic criteria for Alzheimer's disease (AD), reviving the discussion on how to define AD.^1^ This proposal is the latest in a lengthy series of revisions of diagnostic criteria for AD, each evoking renewed discussion on how to define this disease^2^, ^3^, ^4^, ^5^, ^6^, ^7^, ^8^, ^9^, ^10^, ^11^, ^12^, ^13^, ^14^, ^15^, ^16^, ^17^ These criteria elicited a response by a group which is known as the International Working Group (IWG).^18^

The definition of AD has changed numerous times throughout history. Before 1970, AD was regarded as a form of dementia with an early onset.^19^ The first formal diagnostic criteria were published in 1984. These criteria broadened the age criterion to include all ages between 40 and 90. The 1984 criteria were formulated by a working group convened by the National Institute of Neurological and Communicative Disorders and Stroke (NINCDS) and the Alzheimer's Disease and Related Disorders Association (ADRDA).^20^ These initial criteria by the NINCDS‐ADRDA, together with the criteria listed in the respective editions of the Diagnostic and Statistical Manual of Mental Disorders, had been in use for 23 years when the first revision was proposed in 2007.

Since 2007, the diagnostic criteria for AD have been revised nine times by three different groups. The 2007 criteria were formulated by a group of AD researchers that convened as the IWG.^21^ This publication was followed by a series of revisions by the IWG, but also by the National Institute of Aging (NIA) in collaboration with the AA (NIA‐AA), and a collaboration between the IWG and selected members from the AA committees (IWG‐AA). In 2024, the NIA has taken only an advisory role, in line with their general policy toward this kind of scientific work. The revisions implicitly and explicitly reflect a status of the criteria as ‘living documents’ that are to be updated regularly to quickly adapt to the changing field. Most of these successive revisions were intended for use in research practices, while some specifically targeted clinical settings.

The revisions of the AD diagnostic criteria substantially shaped the discussion on the definition of AD. For instance, they introduced novel concepts such as “prodromal AD,” which refers to a state before “full‐blown dementia” in which symptoms are still relatively mild, but biomarkers are abnormal, and “‘preclinical AD,” indicating an asymptomatic phase of the disease with abnormal biomarkers. Moreover, the novel criteria proposed an increasingly biological conception of AD, that is, a disease defined by biological processes rather than symptoms.^1^, ^21^, ^22^, ^23^, ^24^, ^25^, ^26^, ^27^, ^28^, ^29^, ^30^ Such changes have prompted extensive debates in the AD community about whether AD should be defined by biological processes, symptoms, or both.^2^, ^3^, ^4^, ^5^, ^6^, ^7^, ^8^, ^9^, ^10^, ^11^, ^12^, ^13^, ^14^, ^15^, ^16^, ^17^ These debates seem to have resulted in a stalemate between proponents and opponents of biological definitions and/or presymptomatic categories of AD that is closely connected with positions regarding the validity of the amyloid cascade hypothesis.^31^

**RESEARCH‐IN‐CONTEXT**

1. **Systematic review**: We searched the literature on diagnostic criteria for Alzheimer's disease (AD) between 1984 and 2024. Previous studies investigated the implications of one version or a selection of these criteria. We aimed to trace the shifts in aims, addressed problems, and content in the consecutive diagnostic criteria.
2. **Interpretation**: An earlier diagnosis and the incorporation of the latest scientific insights are two recurring aims, but these change in meaning over time. Diagnostic requirements decrease in number, clinical symptoms lose their pivotal role, and amyloid β and tau biomarkers have an increasingly central role, narrowing the conceptualization of AD, and potentially overlooking other important scientific developments.
3. **Future directions**: We propose a more reflective approach to defining AD that starts from an explicit problem and identifies clear aims. Thorough evaluation of the impacts of previous criteria before developing new ones is warranted.

In this article, we aim to constructively contribute to these discussions on the definition of AD and to get beyond the standstill, by providing a longer term view of how diagnostic criteria for AD have evolved. Our aim is to provide insight in how the field has come to where it is and how the different criteria came to be. By critically evaluating these developments, we try to enhance the clinical and scientific debate and include all different viewpoints. We do not aim to come to an exhaustive analysis of the differences between the groups responsible for the revisions. While we partly focus on how the content of the criteria has shifted over time, we first of all seek to trace what motivated subsequent changes. Grasping why (adjustments of) criteria are needed may help to evaluate whether and when revisions are timely and fit for purpose. Accordingly, we systematically analyzed the publications on diagnostic criteria for AD since 1984 and reconstructed (1) the aims, (2) the problems and considerations motivating revision, and (3) the changes in the content of the AD diagnostic criteria.

## 2 METHODS

We identified all diagnostic criteria publications by the NINCDS‐ADRDA, IWG, NIA‐AA, and AA, and searched for reactions or commentaries on these publications between 1984 and 2024. These publications were then systematically analyzed to identify the purported aims of the respective documents, reconstruct the underlying problems and considerations motivating the criteria formulation or revision, and characterize their content.

We used conventional content analysis to identify the aims of the AD diagnostic criteria and reconstruct the problems and considerations that have led to their formulation.^32^ We first highlighted sections from the text that explicitly describe the aims of the authors. These sections were identified by words or word groups that are commonly used to describe aim, such as “we aim to” or “the criteria should be revised to …”. These sections were categorized as “explicit aims” and were coded by their aim (Table S1).

Secondly, we identified sections that referred to considerations and problem descriptions motivating the revisions. These sections were sometimes recognizable by typical words or word groups (such as references to previous criteria), but also included passages describing an apparently problematic situation or developments that novel diagnostic criteria might somehow address. These included, for example, discussions of problems in AD research or of recent scientific insights. These sections were categorized as “problem descriptions and considerations” and were coded according to the type of problem or consideration they were describing (Table S2). Step 1 and 2 were first done by L.v.d.M. and then repeated by M.B. and E.R. Results were compared and discussed to come to a consensus.

Changes in the content of the criteria were assessed by first listing the diagnostic criteria per document. We then systematically compared the contents of each successive document with the previous edition. This led to the identification of specific dimensions of changes, including the number of requirements needed for diagnosis, the types of biomarkers that could be used for diagnosis, and the types of symptoms that were required for diagnosis. The changes in these respective categories were then followed through time, leading to the identification of trends in the evolution of criteria content.

## 3 RESULTS

**FIGURE 1:**
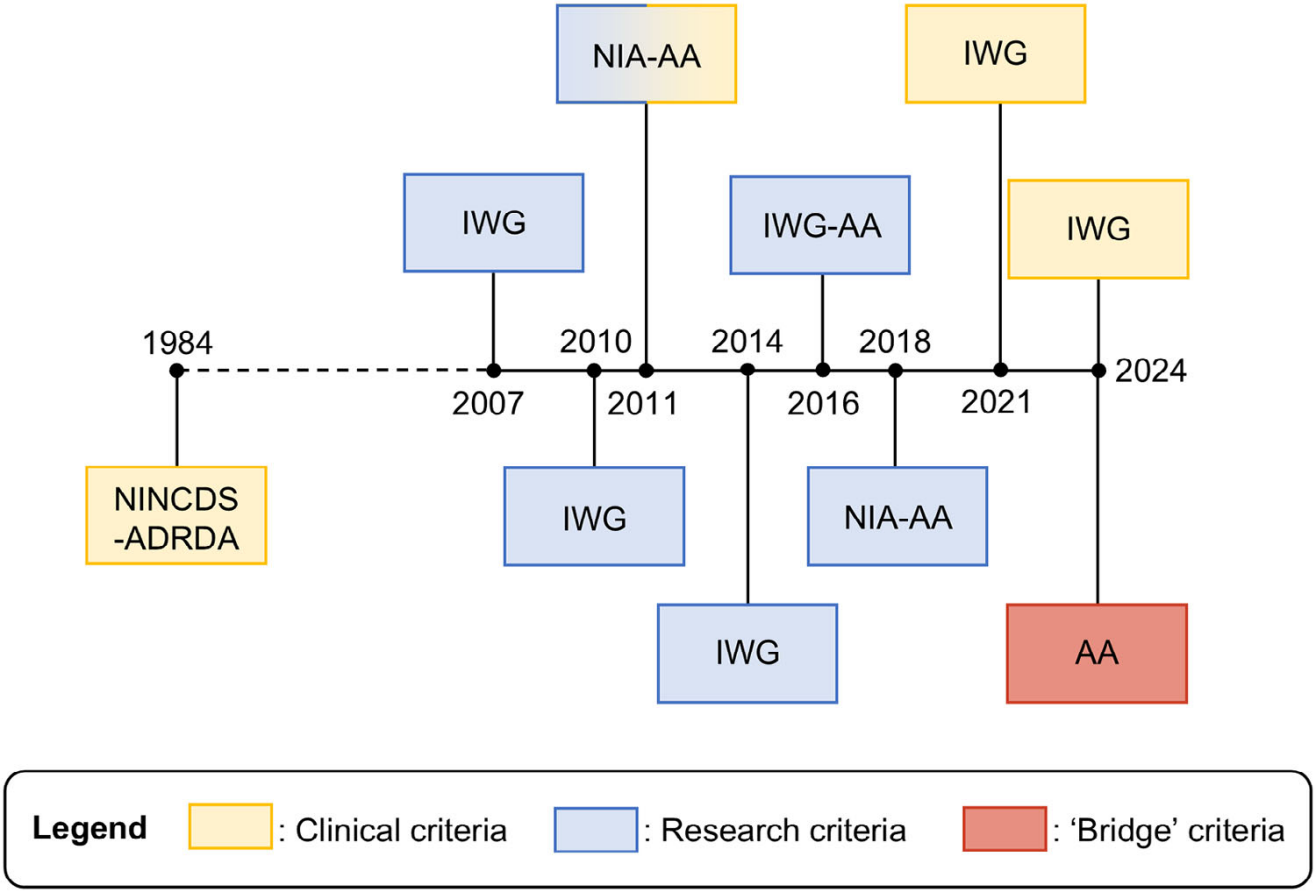
Timeline all published diagnostic criteria for AD. The color of the boxes indicates the intended use of the criteria. AA, Alzheimer's Association; IWG, International Working Group; NIA, National Institute of Aging and the Alzheimer's Association;NINCDS‐ADRDA, National Institute for Neurological and Communicative Disorders and Stroke and the Alzheimer's Disease and Related Disorders Association.

The NINCDS‐ADRDA, IWG, NIA‐AA, and AA produced 10 AD diagnostic criteria versions across a 40‐year timespan. Since 2007, the maximum time between revisions was 3 years (Figure 1). We will subsequently discuss the changes in aims, problem definitions, and content of criteria.

### 3.1 Aims, and problems and considerations motivating the 1984 NINCDS‐ADRDA criteria

In 1984, the NINCDS‐ADRDA committee aimed to formulate diagnostic criteria for AD with particular importance for research and the assessment of the natural history of the disease. On the one hand, this was motivated by the expectation that uniform diagnostic criteria would facilitate the comparison of therapeutic study results. On the other hand, the authors emphasized the need for uniform criteria by referring to the lack of diagnostic accuracy of AD, that is, the discrepancy between the in vivo diagnosis of AD and post‐mortem neuropathological examination of the brain of the diagnosed person (Table 1).^20^

**TABLE 1:** Overview of the explicitly mentioned aims, problem descriptions and considerations, and diagnostic requirements per version of AD diagnostic criteria

AD criteria version	Intended use	Explicitly mentioned aims of the diagnostic criteria	Problem descriptions and considerations motivating diagnostic criteria	Clinical symptoms required for diagnosis	Biomarkers required for diagnosis
NINCDS‐ADRDA 1984	Clinical	‐ Describe clinical criteria for diagnosis of AD of particular importance for research protocols and assessment of the natural history of the disease	‐ Increase diagnostic accuracy ‐ Facilitate comparison between therapeutic trials	‐ Dementia	‐ None required, but “laboratory asssessments” may be used to support an AD diagnosis ‐ Neuropathological evidence of Aβ and tau required for a definite diagnosis
IWG 2007	Research	‐ Incorporate latest scientific insights ‐ Address low specificity of the diagnostic criteria ‐ Enable an early diagnosis of AD to facilitate research on early (prodromal) treatment ‐ Eliminate MCI construct	‐ Definitive diagnosis is only possible post‐mortem ‐ Improved identification of AD phenotype ‐ Lack of clarity in the term “MCI” ‐ Unclear distinction between MCI and AD ‐ New biomarkers for AD	‐ Early and significant progressive episodic memory impairment	‐ MRI MTL atrophy, Abnormal CSF markers for Aβ/tau/future markers, FDG PET or Aβ PET, and/or an autosomal dominant mutation for AD
IWG 2010	Research	‐ Advance and update of 2007 criteria ‐ Provide a lexicon to the AD research community ‐ Enable an early diagnosis of AD to facilitate research on early (prodromal) treatment ‐ Inform clinical field on research developments	‐ Confusion resulting from the dual use of the term “AD” ‐ Unclear characterization of cases with atypical biological or clinical presentation	‐ Early and significant progressive episodic memory impairment, primary progressive non‐fluent aphasia, logopenic aphasia, frontal variant of AD, or posterior cortical atrophy	‐ Pathophysiological markers: CSF markers for Aβ/tau/future markers or Aβ PET ‐ Topographical markers: FDG PET or MRI MTL atrophy
NIA‐AA 2011	Research and clinical	‐ Incorporate latest scientific insights ‐ Enable an early diagnosis to facilitate research on early (preclinical) treatment ‐ Provide a common language for AD researchers ‐ Increase predictive value for clinical outcome	‐ Include better diagnostic distinctions between AD and non‐AD dementias ‐ Neuropathological knowledge on AD and its association with symptoms has increased ‐ No representation of “intermediate” clinical and pathological states in the previous criteria ‐ Unclear distinction between MCI and AD ‐ New biomarkers for AD ‐ No representation of atypical AD in the previous criteria ‐ No representation of genetic factors in the previous criteria ‐ The previous criteria had cutoff values for age ‐ The category of probable AD is too heterogenous	‐ MCI or dementia	‐ Pathophysiological marker: Aβ PET or CSF marker ‐ Neuronal injury marker: Tau CSF, FDG PET, or sMRI
IWG 2014	Research	‐ Present a new algorithm for typical AD ‐ Advance criteria for atypical AD ‐ Refine criteria for mixed AD ‐ Elaborate criteria for preclinical AD	‐ Lack of clarity around the ordering and added value of biomarkers and relationship between biomarkers ‐ Maintaining the principle of high specificity	‐ Early and significant gradual episodic memory impairment	‐ Pathophysiological markers: CSF marker for tau or Aβ, Aβ PET and/or autosomal dominant mutation for AD
IWG‐AA 2016	Research	‐ No concrete objective, goal or aim stated	‐ Absence of standardized definition and methods for preclinical AD	‐ None	‐ PET or CSF marker for tau or Aβ
NIA‐AA 2018	Research	‐ Incorporate latest scientific insights ‐ Formulate a biological definition of AD ‐ Enable an early diagnosis of AD to facilitate research on early (preclinical) treatment ‐ Provide a common language for AD researchers	‐ Confusion resulting from the dual use of the term “AD” ‐ Absence of ordering of biomarkers in previous criteria ‐ Lack of formalization of AD as a ‘continuum’ ‐ Evolution in thinking about biomarkers	‐ None	‐ PET or CSF marker for tau or Aβ
IWG 2021	Clinical	‐ Consider limitations of AD biomarkers for diagnostic use ‐ Provide recommendations on the clinical use of AD biomarkers ‐ Re‐evaluation of a diagnosis solely based on biomarkers	‐ Unclear how to apply biomarkers ‐ Confusion resulting from the dual use of the term “AD” ‐ Limited predictive value of biological definition and presence of pathology for symptoms ‐ Limited knowledge on predictors of symptoms ‐ Problems around validation of cutoff values ‐ Clinical appliance of biomarkers ‐ Ethical problems surrounding biomarker‐based diagnosis of AD.	‐ Amnestic AD, logopenic variant of primary progressive aphasia, posterior cortical atrophy, behavioural or dysexecutive variant, corticobasal syndrome, non‐fluent variant of primary progressive aphasia, and semantic variant of primary progressive aphasia	‐ PET or CSF marker for tau or Aβ
AA 2024	‘Bridge’ between clinical and research	‐ Update 2018 research framework ‐ Incorporate recent advances in biomarker research ‐ Facilitate a bridge between research and clinical care	‐ Biological definition of disease as a general medical standard ‐ Regulatory approval of anti‐Aβ treatments ‐ Development of blood‐based biomarkers ‐ Interchangeability of different means of measuring biomarkers	‐ None	‐ Blood‐based, CSF or PET marker for Aβ and specific forms of phosphorylated and secreted tau
IWG 2024	Clinical	‐ Consider the revised AA criteria ‐ Offer an alternative definition of AD as a clinical‐biological construct for clinical use ‐ Update IWG 2021 criteria	‐ Concerns about the clinical use of a purely biological definition of AD ‐ The understanding of AD by society at large ‐ Translation of blood‐based biomarkers into clinical practice	‐ Amnestic AD, logopenic variant of primary progressive aphasia, posterior cortical atrophy, behavioural or dysexecutive variant, corticobasal syndrome, non‐fluent variant of primary progressive aphasia, and semantic variant of primary progressive aphasia	‐ PET or CSF marker for tau or Aβ, possibly plasma biomarkers in the (near) future

Abbreviations: AA, Alzheimer's Association.; AD, Alzheimer's disease; Aβ, amyloid β; IWG, International Working Group; CSF, cerebrospinal fluid; FDG, fluorodeoxyglucose; MCI, mild cognitive impairment; MRI, magnetic resonance imaging; MTL, medial temporal lobe; NIA, National Institute of Aging and the Alzheimer's Association; NINCDS‐ADRDA, National Institute for Neurological and Communicative Disorders and Stroke and the Alzheimer's Disease and Related Disorders Association; PET, positron emission tomography; sMRI, structural magnetic resonance imaging.

### 3.2 Aims of the IWG and NIA‐AA/AA criteria

The subsequent series of IWG and NIA‐AA/AA criteria justify the need for revision in similar ways. There is substantial overlap between their aims, which is why we discuss these in tandem. Two aims are mentioned by both the IWG and the NIA‐AA/AA, across almost all versions of their AD diagnostic criteria:^1^ to incorporate the latest scientific insights, and^2^ to enable an earlier diagnosis of AD. A third aim,^3^ to provide a common vocabulary for diagnostic practices, is mentioned in some, but not all publications of both the IWG and the NIA‐AA/AA. Lastly, the aim^4^ to increase the accuracy of AD diagnosis, is mentioned by the IWG, only in their first three publications. We discuss these four aims in subsequent order.

Firstly, both the IWG and the NIA‐AA state the aim to incorporate the latest scientific insights into the diagnostic criteria for AD (Table 1). This is often mentioned in both the abstract and the introduction of the publications, indicating their importance. The 2024 AA criteria narrow this aim by referring to advances in biomarker research, rather than scientific advances in general.^1^, ^18^, ^21^, ^22^, ^23^, ^24^, ^25^, ^26^, ^27^, ^28^, ^29^, ^30^

Secondly, both the IWG and NIA‐AA aim to extend the diagnosis of AD into earlier disease stages to enable research on treatments in these stages (Table 1).^20^, ^21^, ^25^, ^26^, ^29^ The rationale mentioned is that the symptoms of AD are presumably caused by the gradual accumulation of amyloid β (Aβ) and hyperphosphorylated tau and that disease‐modifying therapies would be most effective if they are started when protein levels in the brain are still low.^21^, ^26^, ^29^, ^30^

However, the meaning of “an early AD diagnosis” shifts throughout the various editions of the criteria. The 2007 and 2010 IWG criteria refer to the “prodromal” AD phase as being the ideal time to diagnose AD and intervene (Table 1).^21^, ^25^ In the 2011 NIA‐AA criteria, the authors recognize the “preclinical” or “presymptomatic” stadium of AD as the optimal starting point for treatment and thus aim at an earlier diagnosis than that of the preceding IWG criteria.^30^ The subsequent criteria by the IWG, NIA‐AA, and AA continue this trend and refer to the preclinical stage as the ideal moment for diagnosis.^1^, ^26^, ^29^ In contrast, the IWG criteria from 2021 and 2024 argue for an AD diagnosis in the symptomatic stage, based on both biomarkers and symptoms, explicitly defining AD as a clinical‐biological entity. Their main reason for this is that biomarkers are not validated well enough for use in routine clinical diagnostics at a pre‐symptomatic stage.^28^ Thus, the NIA‐AA, AA, and IWG pursue an earlier diagnosis over time, with an exception of the IWG explicitly taking the standpoint that AD should not be diagnosed in the absence of symptoms.

Thirdly, both the IWG and the NIA‐AA aim to provide a common vocabulary for diagnostic practices in AD in their 2010, 2011, and 2018 publications (Table 1).^25^, ^29^, ^30^ The NIA‐AA here specifically intends to provide a common language to AD researchers, while the IWG “lexicon” initially targets research practice, but also anticipates potential use in routine clinical practice. In their most recent publication, the AA presents a variation on this aim by stating that their diagnostic criteria are meant to serve as a “bridge” between research and clinical practice, without explaining why and what this exactly means.^1^

Lastly, the IWG initially aimed for a more precise and accurate diagnosis of AD in their 2007, 2010, and 2014 publications (Table 1).^21^, ^25^, ^26^ The NIA‐AA never denotes a more accurate diagnosis as their aim, but does emphasize the importance of a clearer diagnostic distinction between AD and related dementias.^22^, ^23^ While the IWG does discuss problems on diagnostic accuracy in their publications after 2014, they do not explicitly mention improving the diagnostic accuracy as an aim in these publications.^18^, ^28^

Interestingly, the four aims discussed above are not always explicitly mentioned in the IWG and NIA‐AA/AA publications. The NIA‐AA/AA committees in particular describe problems and considerations that led to the formulation of their criteria, but do not always explain what the novel criteria are supposed to achieve (Table 1). Moreover, the criteria documents also hardly contain any reflection on, for example, the actual (intended or unintended) impact of previous diagnostic criteria since they were published, why the current aims are prioritized over numerous other possible aims, and why some previous aims have been abandoned.^1^, ^18^, ^21^, ^22^, ^23^, ^24^, ^25^, ^26^, ^27^, ^28^, ^29^, ^30^

### 3.3 Problems and considerations motivating the IWG and NIA‐AA/AA criteria

Discussions on what problems and considerations motivated a revision of criteria become less extensive throughout the subsequent IWG and NIA‐AA/AA documents (Table 1). As mentioned above, one of the recurrent aims of the IWG and NIA‐AA/AA criteria revisions is the incorporation of the latest scientific findings. Most of the considerations motivating revision of the criteria are, therefore, research findings. These mainly involve findings from AD biomarker research and not results from the clinical or neuropsychological domain. In particular, the lack of efficient biomarkers and the lack of correct and efficient use of them in practice features in the 2007 IWG criteria and returns in every edition of both groups up until the 2024 criteria. This issue of biomarkers is discussed in various ways. The early documents mainly consider the inclusion of new categories of biomarkers, ranging from Aβ cerebrospinal fluid (CSF) measurements to atrophy in specific brain regions on magnetic resonance imaging (MRI). The considerations in the later documents relate to the hierarchical ordering, the limitations of biomarkers, and the recent advances in research on biomarkers specifically for Aβ and tau.^1^, ^18^, ^21^, ^23^, ^26^, ^28^, ^30^

Moreover, the confusion around the term “AD” prominently features in the publications from 2010 onward (Table 1). This confusion was introduced in the 2007 criteria due to the conceptualization of AD as a “dual clinicobiological entity,” that is, a diagnosis based on both clinical symptoms and pathology, while it was considered to be a symptom‐based diagnosis in the preceding decades.^21^, ^25^ The IWG and the NIA‐AA acknowledge that this dual use of the term “AD” for both the pathology and the set of clinical symptoms may cause misunderstandings and both organizations from 2010 on consistently mention this confusion as being problematic. The IWG even listed this confusion as a pivotal incentive for the publication of their “companion lexicon” in 2010.^18^, ^22^, ^25^, ^26^, ^28^, ^29^ Remarkably, this confusion is not mentioned in the latest criteria document by the AA^1^.

### 3.4 Criteria content

**FIGURE 2:**
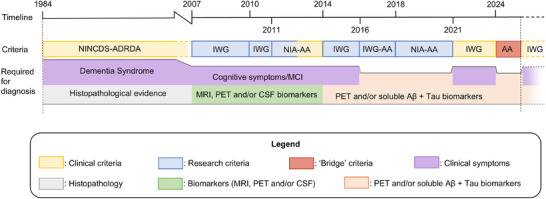
Schematic view of changes in diagnostic requirements for diagnostic criteria by the NINCDS‐ADRDA, IWG, and NIA‐AA/AA. The color of the boxes indicates the intended use of the criteria or the different types of requirements. AA, Alzheimer's Association; Aβ, Amyloid β; CSF, cerebrospinal fluid; IWG, International Working Group; MCI, mild cognitive impairment; MRI, magnetic resonance imaging; NIA‐AA, National Institute of Aging and the Alzheimer's Association; NINCDS‐ADRDA, National Institute for Neurological and Communicative Disorders and Stroke and the Alzheimer's Disease and Related Disorders Association; PET, positron emission tomography.

Subsequent revisions of the diagnostic criteria led to a reduction in the number of requirements needed for an AD diagnosis. This is initially due to a reduction of the number of clinical symptoms required, later a result of relinquishing any clinical symptoms in favor of biomarkers, and most recently the number and type of biomarkers have been reduced (Table 1, Figure 2). We will first discuss the gradual decrease in diagnostic requirements from the 1984 NINCDS‐ADRDA criteria to the subsequent IWG criteria, and, then the shift in requirements from the 1984 NINCDS‐ADRDA criteria to the subsequent NIA‐AA/AA criteria.

The 1984 NINCDS‐ADRDA criteria first of all required the diagnosis of a dementia syndrome, but none of the IWG criteria included this. Rather, the diagnosis of AD according to IWG criteria is based on a combination of memory impairment or other cognitive symptoms only, with, from 2010 onward, the presence of abnormal AD biomarkers with or without symptoms. Furthermore, the age cutoff value and the requirement of histopathological evidence for a definite diagnosis of AD were abandoned from 2007 onward.^20^, ^21^, ^25^, ^26^, ^28^ The IWG introduced two new “preclinical states of AD” in their 2010 criteria and retained these in their subsequent criteria. Firstly, they introduced the “asymptomatic at‐risk state for AD,” an asymptomatic state with abnormal biomarkers for AD. Secondly, they introduced “presymptomatic AD,” an asymptomatic state with an autosomal dominant mutation for AD.^18^, ^25^, ^26^ The authors stress that despite their categorization as “preclinical states of AD,” these categories do not constitute an AD diagnosis.

In the 2011 NIA‐AA criteria, the dementia syndrome is maintained as a requirement for the diagnosis of “AD dementia,” but not for the “preclinical” and “prodromal” states.^22^, ^24^, ^30^ Subsequently, the dementia syndrome is removed from the 2016 IWG‐AA and the 2018 NIA‐AA criteria, which only require abnormal biomarkers for Aβ and tau for an AD diagnosis (Table 1, Figure 2).^27^, ^29^ Indeed, biomarkers for neurodegeneration, that is, MRI measurements for brain atrophy, fluorodeoxyglucose‐positron emission tomography (FDG‐PET) hypometabolism, and CSF tau measures, are also included in the 2018 framework, but are considered neither necessary nor sufficient for an AD diagnosis.^29^ Abnormality of Aβ and tau biomarkers was continued as a sole requirement for the AD diagnosis in the 2024 AA criteria^1^.

Thus, requirements for an AD diagnosis have become less numerous over time in NIA‐AA/AA and IWG criteria, either by replacing the dementia syndrome with cognitive symptoms, or by increasing reliance on biomarkers without the need for symptoms altogether. But the IWG criteria from 2021 and 2024 break with this overall trend of reducing the number of requirements needed for an AD diagnosis, re‐introducing the necessity of clear cognitive symptoms to make a diagnosis of AD (Table 1, Figure 2).^28^

In addition to lowering the diagnostic threshold, the AD diagnostic criteria also increasingly focus on Aβ and tau biomarkers as the most relevant biomarkers (Table 1, Figure 2). In the 1984 NINCDS‐ADRDA criteria, the role of biomarkers (blood, CSF cell count and protein, electroencephalography [EEG], and computed tomography [CT]) was to exclude other diseases.^20^ In contrast, the IWG and NIA‐AA criteria include functional or structural imaging biomarkers, fluid and imaging markers for Aβ and tau, and genetic markers to support the AD diagnosis and confirm the presence of AD pathology.^1^, ^21^, ^23^, ^24^, ^30^ This marks a pivotal change in the function of biomarkers: from a means to exclude other diseases to a parameter to confirm AD. In this manner, biomarkers have taken the role of histopathological examination in the 1984 NINCDS‐ADRDA criteria. Moreover, the range of biomarkers to be used for the diagnosis of AD was further limited to Aβ and tau in the IWG criteria since 2014, and the 2018 and 2024 criteria by the NIA‐AA and the AA.^1^, ^26^, ^28^, ^29^

## 4 DISCUSSION

Our results show that the definition of AD and the criteria for its diagnosis have changed substantially over the years. Two aims are consistently mentioned by the IWG and NIA‐AA/AA:^1^ enabling an early diagnosis to facilitate early therapeutic trials and^2^ incorporating the latest scientific advances. The implications of these results will be discussed in more depth, and subsequently, we will provide an account to aid in overcoming their potential limitations.

The meanings of the two central aims are changing over time. Firstly, early diagnosis becomes ever earlier over time, including “prodromal” and “preclinical” phases. Secondly, the scope of scientific developments incorporated in the criteria becomes narrower over time, increasingly focusing on biomarker research and less on neuropsychological or clinical studies. This suggests that the scope of scientific developments was determined by the aim to facilitate early diagnosis. Moreover, the number of discussed problems and considerations has decreased. This could either signify that there are simply fewer developments to be incorporated (which would cast doubt on the need for revision), or that other developments are ignored without explaining why this would be justified. On a more general note, the status of the criteria as “living documents” that are updated regularly allows for quick incorporation of scientific findings, but it is not evident that novel scientific insights in themselves justify disease criteria revisions. Thorough reflection on the previous criteria, their goals and practical implementation, and the added value of new findings to these criteria should precede any update of diagnostic criteria.

Our analysis shows that the confusion surrounding the term ‘AD’ is persistently presented as a problem to be addressed since 2010. Whereas AD had been used to refer to a clinical phenomenon, the IWG in 2007 and NIA‐AA in 2011 started using it for the associated pathology as well, making the term more ambiguous. The differences in intended use designations of the consecutive diagnostic criteria further contributed to the confusion. Rather than providing the AD community with a clear lexicon or common language, the revisions have increased confusion on what AD entails.

Both the aims and the criteria content are increasingly subject to “biomarkerization,” that is, are increasingly shaped by biomarkers.^33^ Together, the changes in criteria content allow for an “early” AD diagnosis before the stage of “full‐blown” dementia, and, in the case of the 2024 AA criteria, before the onset of symptoms. This finding aligns with previous observations noting a trend toward a biological definition of AD, as well as a drive toward an ever earlier diagnosis.^19^, ^34^, ^35^, ^36^, ^37^ The desirability of these developments has been contested. On the one hand, biomarker‐based diagnosis may have beneficial effects, allowing for early intervention or prevention. A biomarker‐based diagnosis of AD allows for treatment in presymptomatic or prodromal phases of the disease, if such treatments become available with an adequate evidence‐base. Furthermore, the emergence of efficient treatments targeting other proteinopathies may necessitate biomarkers to identify the appropriate patients for these therapies. Such scenarios currently remain hypothetical and a thorough analysis is still needed to identify and address the ethical and implementation issues. On the other hand, a biomarker‐based diagnosis may lead to an expansion of the number of people diagnosed with AD, some of whom may never experience any symptoms or remain asymptomatic for many years,^19^, ^34^, ^37^, ^38^, ^39^, ^40^, ^41^, ^42^ a situation that the IWG explicitly aims to avoid in the 2021 and 2024 criteria.

It could be argued that these drawbacks mainly concern the clinical use of the criteria, whereas most of the diagnostic criteria are primarily intended for use in research settings. Although AD research may benefit from a biomarker‐based diagnosis, the criteria revisions may also have strong, potentially detrimental, implications for AD research. Firstly, the high frequency of criteria revisions may cause different studies to use different criteria, complicating the comparison of research results (the opposite of what the NINCDS‐ADRDA aimed to achieve with the 1984 criteria).

Secondly, the increasing focus on Aβ and tau as central diagnostic biomarkers may limit research on other possible mechanisms in AD.^29^ Such a reduction of scope is problematic, especially since the centrality of Aβ and tau in the pathogenesis of AD is a matter of debate.^7^, ^8^, ^9^, ^14^, ^15^ Studies on the societal impact of science and technology have shown that the very emergence of conventions like diagnostic criteria already validates the use of the objects they concern, potentially contributing to a self‐fulfilling prophecy.^43^ Thus, the increasing centrality of Aβ and tau in the diagnostic criteria may lead to AD research favoring these two biomarkers, regardless of their validation status and reassurances that this is not the intention.^17^

Discussions about the desirability of a biological definition of AD often result in a stalemate. The same is actually true for more general philosophical discussions on the conceptualization of disease.^44^, ^45^, ^46^, ^47^ Recently, a pragmatic approach to defining health and disease was proposed as a way out of the philosophical polarization. This approach holds that a definition of disease should fit with the context in which it is used and the aims to be achieved. For instance, one definition of disease may be suited for selecting specific clinical trial participants but not for treating a heterogeneous patient group in the clinic. An important implication of this approach is that changes in disease definitions may very well be justified if it is clear why these changes occur and what problem they should address.^48^ In the case of the AD diagnostic criteria revisions, however, aims are often not explicitly mentioned. The methods to achieve these aims are also not described by the NIA‐AA/AA, and only sparsely by the IWG. Moreover, reflection on the reasons for changes in aims, the consequences of the previous revisions, or the progress toward the present or previous aims is limited or outright absent.

We found hardly any evidence of committees responding to criticism brought forward to previous criteria. As a result, arguments mentioned in commentaries on the earliest publications are brought forward again in response to later criteria.^3^, ^7^, ^8^, ^9^, ^14^, ^15^ For instance, both the 2010 IWG criteria and the 2018 NIA‐AA criteria have been referred to as a “Tower of Babel” by different commentators.^7^, ^8^ Also, the composition of the criteria committees has been criticized repeatedly.^2^, ^3^, ^19^ Despite these critiques, a substantial number of authors on the diagnostic criteria of all groups report interests with pharmaceutical companies and the number of pharmaceutical company employees is growing in the committees of the NIA‐AA and the AA. These findings align with a recent publication describing the scientific dialogue in the AD field as being stuck in a “Groundhog Day scenario”: a circular loop with no apparent breakthrough on the horizon.^31^

Interestingly, the 2021 and 2024 IWG criteria demonstrate how a change in the intended context of use (from research to clinic) led to reflections on the limitations of the biological disease definition proposed previously. A similar reflective attitude would be warranted, however, for any context of use.

How then, to move beyond the present “Groundhog Day scenario”’? Our long‐term view suggests that committees considering whether and, if so, how to revise AD diagnostic criteria should start with clearly identifying and elaborating the problems that the criteria revisions aim to address, as well as who experiences these problems. These committees should be more diverse with broader expertise and less industry involvement. The committee should evaluate the implications of the previous revision in their broadest sense and the progress made toward the intended aims. This would allow criteria committees to build on the unmet goals of previous revisions and explicitly formulate new goals to meet the limitations of preceding criteria. A previously developed checklist with considerations for redefining a disease could be used.^49^ Our reconstruction suggests that such an approach is desirable to overcome the challenges we identified.

A limitation of our study is its focus on diagnostic criteria publications and related documents. We cannot exclude the possibility that the aims of the diagnostic criteria revisions were described differently or that response to criticism may have happened elsewhere. Nevertheless, we think that the academic debate on the definition of AD would benefit from maximal transparency and accessibility of reasons underlying criteria revisions. Diagnostic criteria publications should therefore start from an explicit problem and aim statement and include reflections on the aims of previous criteria, their implications, and the reactions they evoked.

In conclusion, the two main aims of the NIA‐AA/AA and IWG diagnostic criteria are an earlier diagnosis of AD and the incorporation of the latest findings from biomarker research. These two in tandem have led to an increasing biomarkerization of the AD concept which may have substantial effects on AD research and the dementia field as a whole. However, explicit justification of these aims and the revisions they informed is often lacking in criteria documents. A more reflective approach with clear aims and consistent evaluation of the impacts of previous criteria is needed to advance discussions on how to best define and diagnose AD.

## CONFLICT OF INTEREST STATEMENT

Author disclosures are available in the supporting information.

## CONSENT STATEMENT

No consent was necessary.

## Supporting information



Supporting Information

Supporting Information
