# Supplementary material for: Changing definitions of disease: Transformations in the diagnostic criteria for Alzheimer's disease
Source: Alzheimers Dement. 2025 Apr 12;21(4):e70133. doi: 10.1002/alz.70133 (PMC11992595; doi:10.1002/alz.70133)
Supplement: Supplementary file 1 — Supporting Information [file ALZ-21-e70133-s002.docx]

**Table S1: Overview of exemplary quotes per explicit aim of diagnostic criteria document**

| **AD criteria version** | **Explicit aim** | **Exemplar Quote** |
| --- | --- | --- |
| NINCDS-ADRDA 1984 | 1. Describe clinical criteria for diagnosis of AD of particular importance for research protocols and assessment of the natural history of the disease | 1. *“*The group intended to establish and to describe clinical criteria for the diagnosis of Alzheimer's disease of particular importance for research protocols and to describe approaches that would be useful for assessing the natural history of the disease.” (p.939) |
| IWG 2007 | 1. Incorporate latest scientific insights 2. Address low specificity of the diagnostic criteria 3. Enable an early diagnosis of AD to facilitate research on early (prodromal) treatment 4. Eliminate MCI construct | 1. “The NINCDS–ADRDA and the DSM-IV-TR criteria for Alzheimer’s disease (AD) are the prevailing diagnostic standards in research; however, they have now fallen behind the unprecedented growth of scientific knowledge.” (p. 734) 2. “Nevertheless, the low specificity must be addressed through both revised AD and accurate non-AD dementia diagnostic criteria.” (p. 735) 3. “Revised research criteria would allow diagnosis when symptoms first appear, before full-blown dementia, thus supporting earlier intervention at the prodromal stage.” (p. 736) 4. “Our proposal for multidimensionally established identification of AD would have potential superiority to the intrinsically heterogeneous state of mild cognitive impairment and would advance the concept of mild cognitive impairment to its natural next level of more desirably identifying prodromal AD.” (p. 736) |
| IWG 2010 | 1. Advance and update of 2007 criteria 2. Provide a lexicon to the AD research community 3. Enable an early diagnosis of AD to facilitate research on early (prodromal) treatment 4. Inform clinical field on research developments | 1. “The aim of this paper is to advance the new research criteria initiative [2007 diagnostic criteria] by providing a companion lexicon, wherein the different entities and concepts related to AD are defined and updated.” (p.1118) 2. “This lexicon for AD is primarily intended to serve the research community by providing a framework of the disease that covers its full spectrum, and which should be used for research protocols and clinical trials directed at early intercession in the pathogenic cascade of the disease.” (p.1118) 3. “The secondary aim of the paper is to provide clinicians with a clear view of this evolving field [research on early stages of AD and disease-modifying treatments] in which use of biomarkers is advancing and might reach regulatory qualifications and approval in the foreseeable future. These dual aims are intended to keep the research and clinical view of the disease from becoming too widely separated.” (p.1118) |
| NIA-AA 2011 | 1. Incorporate latest scientific insights 2. Enable an early diagnosis to facilitate research on early (preclinical) treatment 3. Provide a common language for AD researchers 4. Increase predictive value for clinical outcome | 1. “Ultimately, it is hoped that the scientific knowledge gained over the past quarter of a century, leading to the reconceptualization of “Alzheimer’s disease” proposed by the NIA-Alzheimer’s Association workgroup, will result in improved diagnosis and ultimately in effective disease-modifying therapy.” (p.260) 2. “It is hoped that these recommendations will provide a common rubric to advance the study of preclinical AD, and ultimately, aid the field in moving toward earlier intervention at a stage of AD when some disease-modifying therapies may be most efficacious.” (p. 281) 3. “Furthermore, we propose a research framework to provide a common language to advance the scientific understanding of the preclinical stages of AD and a foundation for the evaluation of preclinical AD treatments.” (p. 281) 4. “For these reasons, our working group sought to examine the evidence for a definable preclinical stage of AD, and to review the biomarker, epidemiological, and neuropsychological factors that best predict the risk of progression from asymptomatic to MCI and AD dementia.” (p. 281) |
| IWG 2014 | 1. Present a new algorithm for typical AD 2. Advance criteria for atypical AD 3. Refine criteria for mixed AD 4. Elaborate criteria for preclinical AD | “Our aims are as follows:  (1) to present a new diagnostic algorithm for typical AD;  (2) to advance the diagnostic criteria for atypical AD;  (3) to refine the diagnostic criteria for mixed AD;  (4) to elaborate the criteria for the diagnosis of the preclinical states of AD;  and (5) to differentiate the biomarkers of AD diagnosis from those of AD progression.” (p. 614) |
| IWG-AA 2016 | No concrete objective, goal or aim stated |  |
| NIA-AA 2018 | 1. Incorporate latest scientific insights 2. Formulate a biological definition of AD 3. Enable an early diagnosis of AD to facilitate research on early (preclinical) treatment 4. Provide a common language for AD researchers | 1. “Scientific progress in the interim led to an initiative by the National Institute on Aging and Alzheimer’s Association to update and unify the 2011 guidelines.” (p. 536) 2. “Thus, a framework suitable for interventional trials should be founded on a biologically based definition of AD; and, it is only rational that the framework is harmonized across interventional and observational research.” (p. 536) 3. “Furthermore, in order to discover interventions that prevent or delay the initial onset of symptoms a biologically based definition of the disease that includes the preclinical phase is needed.” (p. 536) 4. “The framework described in this document also has this latter intention—to provide researchers a common language with which to communicate observations.” (p. 536) |
| IWG 2021 | 1. Consider limitations of AD biomarkers for diagnostic use 2. Provide recommendations on the clinical use of AD biomarkers 3. Re-evaluation of a diagnosis solely based on biomarkers | 1. “Increased accessibility to biomarkers, and the potential for blood biomarkers to provide information about the underlying disease processes in the future, necessitate consideration of the limitations of biomarkers in the diagnosis of Alzheimer’s disease, and recommendations about how these biomarkers should and should not be used in a clinical setting.” (p. 484) 2. “3 years after introduction of the NIA-AA criteria, a re-evaluation of a diagnostic approach based only on biological markers is warranted for both conceptual and evidence-based practical reasons.” (p. 484-485) |
| AA 2024 | 1. Update 2018 research framework 2. Incorporate recent advances in biomarker research 3. Facilitate a bridge between research and clinical care | 1. “The present document updates the 2018 research framework in response to several recent developments.” (p. 1) 2. “Our intent is to present objective criteria for diagnosis and staging AD, incorporating recent advances in biomarkers, to serve as a bridge between research and clinical care.” (p. 2) 3. “We therefore view these criteria as a bridge between research and clinical care.” (p. 3) |
| IWG 2024 | 1. Consider the revised AA criteria 2. Offer an alternative definition of AD as a clinical-biological construct for clinical use 3. Update IWG 2021 criteria | 1. “To consider the revised AA criteria and to offer an alternative definitional view of AD as a clinical-biological construct for clinical use.” (p. E1) 2. “The recommendations of the 2021 IWG diagnostic criteria are updated for further elaborating at-risk and presymptomatic states.” (p. E1) |

Abbreviations: NINCDS-ADRDA, National Institute for Neurological and Communicative Disorders and Stroke and the Alzheimer’s Disease and Related Disorders Association; AD, Alzheimer’s Disease; IWG, International Working Group; MCI, Mild Cognitive Impairment; DSM-IV-TR, Diagnostic and Statstical Manual for Psychiatric Disorders; NIA, National Institute of Aging and the Alzheimer’s Association; AA, Alzheimer’s Association.

**Table S2: Overview of exemplary quotes per problem description and consideration of diagnostic criteria document**

| **AD criteria version** | **Problem descriptions and considerations** | **Exemplar Quote** |
| --- | --- | --- |
| NINCDS-ADRDA 1984 | 1. Increase diagnostic accuracy 2. Facilitate comparison between therapeutic trials | 1. “The need to refine clinical diagnostic criteria has been emphasized because 20% or more of cases with the clinical diagnosis of Alzheimer’s disease are found at autopsy to have other conditions and not Alzheimer’s disease.” (p. 939) 2. “Moreover, therapeutic trials can be meaningfully compared only if uniform criteria are used for diagnosis and response to treatment.” (p. 939) |
| IWG 2007 | 1. Definitive diagnosis is only possible post-mortem 2. Improved identification of AD phenotype 3. Unclarity surrounding the term ‘MCI’ 4. Unclear distinction between MCI and AD 5. New biomarkers for AD | 1. “The currently accepted criteria support a probabilistic diagnosis of AD within a clinical context where there is no definitive diagnostic biomarker. A definite diagnosis of AD is only made according to the NINCDS–ADRDA criteria when there is histopathological confirmation of the clinical diagnosis.” (p. 734) 2. “When the NINCDS–ADRDA criteria were first published, the authors noted that they were not yet fully operational because of insufficient knowledge about the disease. Since then, the clinical phenotype of AD has been much more clearly elucidated.” (p. 735) 3. “A series of large randomised controlled trials with both non-steroidal anti-inflammatory drugs and acetylcholinesterase inhibitors have sought to establish the usefulness of these drugs in delaying the conversion of mild cognitive impairment to AD. However, the lessons learned have highlighted the problems of mild cognitive impairment within this type of randomised controlled trial” (p. 736) 4. “The transition from mild cognitive impairment to AD has been an a priori primary endpoint in several randomised controlled trials. There is an inherent arbitrariness in determining a binary outcome, that is, conversion or no conversion, when the underlying disease is a continuous process.” (p. 736-737). 5. “Over the past two decades since the NINCDS–ADRDA criteria were published, great progress has been made in identifying the AD-associated structural and molecular changes in the brain and their biochemical footprints.” (p. 737) |
| IWG 2010 | 1. Confusion resulting from the dual use of the term ‘AD’ 2. Unclear characterization of cases with atypical biological or clinical presentation | 1. “(…) the proposal of a “dual clinicobiological entity” that can be diagnosed during life also raises new questions about the definition of AD. For example, this framework did not initially address the nosology of AD-related states if the defining clinicobiological duality is not present. Additionally, conditions still to be considered within the new research criteria framework include the nosological classification of clinically asymptomatic individuals who are positive for biomarkers of Alzheimer’s pathology, clinically symptomatic individuals without evidence of biomarker findings, or those with atypical features (atypical AD). There are now increasingly well recognised atypical presentations that include non-amnestic focal cortical syndromes, such as progressive non-fluent aphasia, logopenic aphasia, and posterior cortical atrophy, that are confirmed neuropathologically as being AD.” (p. 1118) |
| NIA-AA 2011 | 1. Include better diagnostic distinctions between AD and non-AD dementias 2. Neuropathological knowledge on AD and its association with symptoms has increased 3. No representation of ‘intermediate’ clinical and pathological states in the previous criteria 4. Unclear distinction between MCI and AD 5. New biomarkers for AD 6. No representation of atypical AD in the previous criteria 7. No representation of genetic factors in the previous criteria 8. The previous criteria had cut-off values for age 9. The category of probable AD is too heterogenous | 1. “Because knowledge of the non-AD dementias was considerably more rudimentary in 1984, the 1984 criteria were vague in defining how distinctions between AD dementia and the major alternatives should be made.” (p. 259) 2. “When the NINCDS–ADRDA criteria were formulated, it was believed that AD, like many other brain diseases, always exhibited a close correspondence between clinical symptoms and the underlying pathology, such that (1) AD pathology and clinical symptoms were synonymous, and (2) individuals either had fully developed AD pathology, in which case they were demented, or they were free of AD pathology, in which case they were not demented (at least not because of AD). However, in the intervening 27 years, it has become clear that this clinical-pathological correspondence is not always consistent.” (p. 258) 3. “The 1984 criteria did not account for cognitive impairment that did not reach the threshold for dementia. The 1984 one-to-one clinical-pathological correspondence model did not account for the fact that AD-P develops slowly over many years, with dementia representing the end stage of many years of pathology accumulation in those patients who do become demented. Moreover, intermediate levels of AD pathological severity map onto clinical impairment, which is intermediate between normality and dementia.” (p. 259) 4. “It is important to note that, as AD is a slow, progressive disorder, with no fixed events that define its onset, it is particularly challenging for clinicians to identify transition points for individual patients. Thus, the point at which an individual transitions from the asymptomatic phase to the symptomatic predementia phase [3], or from the symptomatic predementia phase to dementia onset, is difficult to identify [2]. Moreover, there is greater diagnostic uncertainty earlier in the disease process. It is, nevertheless, important to incorporate this continuum of impairment into clinical and research practice.” (p. 271) 5. “No inclusion of results of magnetic resonance imaging, positron emission tomography (PET) imaging, and cerebrospinal fluid (CSF) assays (that we will refer to subsequently as biomarkers) in decision-making. Initial efforts to incorporate biomarkers into the diagnosis of AD dementia and MCI [14] need to be coupled with a more comprehensive approach to the diagnostic process.” (p. 264) 6. “The implication that memory impairment is always the primary cognitive deficit in all patients with AD dementia. Experience has shown that there are several nonamnestic presentations of the pathophysiological process of AD” (p. 264) 7. “Lack of information about the genetics of AD” (p. 264) 8. “Proposed age cutoffs for the diagnosis of AD dementia. Work over the past decades has established that AD dementia in those aged ,40 years, although rare, does not differ in its pathophysiology from older persons.” (p. 264) 9. “Extreme heterogeneity of the “Possible” AD dementia category, including a group of patients who would now be diagnosed as “Mild cognitive impairment (MCI).” (p. 264) |
| IWG 2014 | 1. Unclarity around the ordering and added value of biomarkers and relationship between biomarkers 2. Maintaining the principle of high specificity | 1. “The most challenging questions for both the IWG and NIA–AA frameworks focus on the multitude of proposed biomarkers, their inter-relationships particularly with regard to producing additive value, and their putative weight in the diagnosis. A temporal order has been proposed in the NIA–AA research criteria for preclinical AD, in which amyloid biomarker changes precede neuronal injury in the progression to symptomatic stages. This ordering is not settled, although it has received some support from the cross-sectional baseline data of genetically identified early-onset familial AD cases with various presenilin and amyloid precursor protein mutations” (p. 616) 2. “[…]maintaining the principle of a high specificity.” (p. 614) |
| IWG-AA 2016 | 1. Absence of standardized definition and methods for preclinical AD | 1. “The great heterogeneity of methodologies used in different studies referring to different definitions of preclinical AD has created confusion. Standardizing of these definitions is important to future AD research.” (p. 294) |
| NIA-AA 2018 | 1. Confusion resulting from the dual use of the term ‘AD’ 2. Absence of ordering of biomarkers in previous criteria 3. Lack of formalization of AD as a ‘continuum’ 4. Evolution in thinking about biomarkers | 1. “Consequently, the term AD is often used to describe two very different entities: prototypical clinical syndromes without neuropathologic verification and AD neuropathologic changes. However, a syndrome is not an etiology but rather a clinical consequence of one or more diseases. A biological rather than a syndromal definition of AD is a logical step toward greater understanding of the mechanisms underlying its clinical expression. Disease-modifying interventions must engage biologically defined targets, and the dementia syndrome does not denote a specific biological target(s). Furthermore, in order to discover interventions that prevent or delay the initial onset of symptoms a biologically based definition of the disease that includes the preclinical phase is needed. Thus, a framework suitable for interventional trials should be founded on a biologically based definition of AD; and, it is only rational that the framework is harmonized across interventional and observational research.” (p. 536) 2. “In the 2011 recommendations, amyloid biomarkers were placed at the apex of the biomarker hierarchy preclinically, whereas in contrast, all AD biomarkers, including those reflecting neurodegeneration, were placed on equal footing in the MCI and dementia guidelines. Although this discrepancy was noted at the time, there is now a growing consensus that application of biomarkers should be harmonized conceptually across the disease continuum and that biomarkers of neurodegeneration are not equivalent to those reflecting amyloid and pathologic tau accumulation .” (p. 537) 3. “Thus, the disease is now regarded as a continuum rather than three distinct clinically defined entities. This concept was recognized but was not formalized in the 2011 NIA-AA guidelines.” (p. 537) 4. “A major motivation for updating the 2011 guidelines has been the evolution in thinking about biomarkers. Studies published since 2011 have reinforced the idea that certain imaging and CSF biomarkers are valid proxies for neuropathologic changes of AD.” (p. 537) |
| IWG 2021 | 1. Unclarity about efficient appliance of biomarkers 2. Confusion resulting from the dual use of the term ‘AD’ 3. Limited predictive value of biological definition and presence of pathology for symptoms 4. Limited knowledge on predictors of symptoms 5. Problems around validation of cut-off values 6. Clinical appliance of Biomarkers 7. Ethical problems surrounding biomarker-based diagnosis of AD. | 1. “Increased accessibility to biomarkers, and the potential for blood biomarkers to provide information about the underlying disease processes in the future, necessitate consideration of the limitations of biomarkers in the diagnosis of Alzheimer’s disease, and recommendations about how these biomarkers should and should not be used in a clinical setting.” (p. 484) 2. “Based on ATN status, Alzheimer’s disease could be considered as a purely biological condition, dissociated from a clinical component or individual status. By dissociating Alzheimer’s disease from a clinical phenotype, the disease instead equates to Alzheimer’s disease neuropathological changes, whereas in 2012, neuropathologists stated that “There is consensus to disentangle the clinicopathologic[al] term ‘Alzheimer’s disease’ from [Alzheimer’s disease] neuro pathologic[al] change” (p. 485) 3. “A major limitation of a purely biological definition of Alzheimer’s disease is its low predictive accuracy.” (p. 485) 4. “In summary, amyloid β and tau biomarkers are not sufficient to confidently predict progression to prodromal Alzheimer’s disease or Alzheimer’s disease dementia, or to define a person’s position on the Alzheimer’s disease continuum, without clinical input.” (p. 487) 5. “Most of all, this binary threshold does not reflect the reality of amyloid β and tau pathology, which is continuous and present at a minimal extent in almost all people older than 70 years, with important discrepancies between pathology burden and clinical symptoms at intermediate extents” (p. 487) 6. “Considering general medical practice and standard of care, the six currently available Cochrane reviews on the use of CSF or amyloid PET biomarkers have consistently led to the same conclusion: that the routine use of these biomarkers in clinical practice cannot be recommended” (p. 489) 7. “Informing cognitively unimpaired individuals that they have an irreversible disease on the basis of biomarkers is ethically challenging, given that the clinical trajectory towards prodromal Alzheimer’s disease or Alzheimer’s disease dementia is uncertain, and that there is no way to prevent the development of symptoms in the absence of modifiable risk factors or specific therapies.” (p. 490) |
| AA 2024 | 1. Biological definition of disease as a general medical standard 2. Regulatory approval of anti-Aβ treatments 3. Development of blood-based biomarkers 4. Interchangeability of different means of measuring biomarkers | 1. “Defining diseases biologically, rather than based on syndromic presentation, has long been standard in many areas of medicine (e.g., oncology), and is becoming a unifying concept common to all neurodegenerative diseases, not just AD.” (p. 2) 2. “First, treatments that target core disease pathology have, for the first time, received regulatory approval.” (p. 3) 3. “Second, the most significant advance in AD diagnostics in recent years has been the development of blood-based markers (BBM) with some (not all) assays exhibiting accurate diagnostic performance.” (p. 3) 4. “Finally, an important product of recent research is the recognition that imaging, CSF, and BBM within a pathobiological AT(N) (amyloid/tau/neurodegeneration) category are interchangeable for some, but not all, intended uses.” (p. 3) |
| IWG 2024 | 1. Concerns about the clinical use of a purely biological definition of AD 2. The understanding of AD by society at large 3. Translation of blood-based biomarkers into clinical practice | 1. “However, concerns about a purely biological definition of AD being applied clinically, the understanding of AD by society at large, and the translation of blood-based biomarkers into clinical practice prompt these International Working Group (IWG) updated recommendations.” (p. E1) |

Abbreviations: NINCDS-ADRDA, National Institute for Neurological and Communicative Disorders and Stroke and the Alzheimer’s Disease and Related Disorders Association; IWG, International Working Group; AD, Alzheimer’s Disease; MCI, Mild Cognitive Impairment; PET, Positron Emission Tomography; CSF, Cerebrospinal Fluid; NIA, National Institute of Aging and the Alzheimer’s Association; AA, Alzheimer’s Association.
